# Activation of Focal Adhesion Kinase Restores Simulated Microgravity-Induced Inhibition of Osteoblast Differentiation via Wnt/Β-Catenin Pathway

**DOI:** 10.3390/ijms23105593

**Published:** 2022-05-17

**Authors:** Cuihong Fan, Zhaojia Wu, David M. L. Cooper, Adam Magnus, Kim Harrison, B. Frank Eames, Rajni Chibbar, Gary Groot, Junqiong Huang, Harald Genth, Jun Zhang, Xing Tan, Yulin Deng, Jim Xiang

**Affiliations:** 1 Cancer Research, Saskatchewan Cancer Agency, Saskatoon, SK S7N 4H4, Canada; chfan@bit.edu.cn (C.F.); zhaojia.wu@usask.ca (Z.W.); 2 Department of Oncology, University of Saskatchewan, Saskatoon, SK S7N 5E5, Canada; 3 School of Life Sciences, Beijing Institute of Technology, Beijing 100811, China; zhangjun@bit.edu.cn (J.Z.); tanxing@bit.edu.cn (X.T.); deng@bit.edu.cn (Y.D.); 4 Department of Anatomy, Physiology and Pharmacology, University of Saskatchewan, Saskatoon, SK S7N 5E5, Canada; david.cooper@usask.ca (D.M.L.C.); adm426@usask.ca (A.M.); kharrison226@hotmail.com (K.H.); brian.eames@usask.ca (B.F.E.); 5 Department of Pathology, University of Saskatchewan, Saskatoon, SK S7N 5E5, Canada; rajni.chibbar@saskhealthauthority.ca; 6 Department of Surgery, Royal University Hospital, University of Saskatchewan, Saskatoon, SK S7N 5E5, Canada; gary.groot@usask.ca; 7 Department of Blood Transfusion, Affiliated Hospital of Zunyi Medical University, Zunyi 563006, China; junqiongh@aliyun.com; 8 Institute of Toxicology, Hannover Medical School, D-30625 Hannover, Germany; genth.harald@mh-hannover.de

**Keywords:** SMG, FAK, Wnt/β-catenin, osteoblast, ALP activity, mineralization, bone density, micro-CT, CNF1, hindlimb unloading model

## Abstract

Simulated microgravity (SMG) inhibits osteoblast differentiation (OBD) and induces bone loss via the inhibition of the Wnt/β-catenin pathway. However, the mechanism by which SMG alters the Wnt/β-catenin pathway is unknown. We previously demonstrated that SMG altered the focal adhesion kinase (FAK)-regulated mTORC1, AMPK and ERK1/2 pathways, leading to the inhibition of tumor cell proliferation/metastasis and promoting cell apoptosis. To examine whether FAK similarly mediates SMG-dependent changes to Wnt/β-catenin in osteoblasts, we characterized mouse MC3T3-E1 cells cultured under clinostat-modeled SMG (µg) conditions. Compared to cells cultured under ground (1 g) conditions, SMG reduces focal adhesions, alters cytoskeleton structures, and down-regulates FAK, Wnt/β-catenin and Wnt/β-catenin-regulated molecules. Consequently, protein-2 (BMP2), type-1 collagen (COL1), alkaline-phosphatase activity and matrix mineralization are all inhibited. In the mouse hindlimb unloading (HU) model, SMG-affected tibial trabecular bone loss is significantly reduced, according to histological and micro-computed tomography analyses. Interestingly, the FAK activator, cytotoxic necrotizing factor-1 (CNF1), significantly suppresses all of the SMG-induced alterations in MC3T3-E1 cells and the HU model. Therefore, our data demonstrate the critical role of FAK in the SMG-induced inhibition of OBD and bone loss via the Wnt/β-catenin pathway, offering FAK signaling as a new therapeutic target not only for astronauts at risk of OBD inhibition and bone loss, but also osteoporotic patients.

## 1. Introduction

Spaceflight contains many physiological, environmental and psychological stress factors that seriously affect the health of astronauts [1]. One of these stress factors is aerospace microgravity (AMG), which has been demonstrated to cause alterations in various biological systems, resulting in cardiovascular and immune disorders and atrophy of the muscular and skeletal systems [1,2]. One of the most significant physiological challenges astronauts face is AMG-induced osteoblast differentiation (OBD) inhibition and bone loss. Simulated microgravity (SMG), a ground-based method developed to mimic AMG microgravity conditions, was developed to study the effects and molecular mechanisms of AMG-altered OBD. It has been demonstrated that SMG suppresses osteoblast differentiation via the Wnt/β-catenin pathway in a similar manner to patients with osteoporosis [3,4]. However, the mechanism through which SMG alters the Wnt/β-catenin pathway is unknown.

The Wnt/β-catenin pathway has an important role in OBD and, thus, in the formation and regeneration of bone [3]. Wnt signaling down-regulates the activity of glycogen synthase kinase-3β (GSK3β), leading to the hypo-phosphorylation of β-catenin [3]. The stabilized β-catenin aggregates in the cytosol and translocates into the nucleus [5]. This results in an interaction with the transcription factors T cell factor-1 (TCF1) and lymphoid enhancer binding factor (LEF), which further up-regulates the osteoblast maturation markers bone morphogenic protein-2 (BMP2) and type-1 collagen (COL1), thus mediating the Wnt’s effect on the osteoblast maturation characteristics measured by alkaline phosphatase (ALP) activity and mineralization analyses [3,6,7].

Cytoskeleton structures play important roles in cell biology by maintaining cells’ shapes, providing a cell transport system and controlling cell migration and survival [8]. The eukaryotic cytoskeleton is composed of actin and intermediate filaments and microtubules. Cell surface integrins interact with the extracellular matrix at cellular membrane sites, called focal adhesions (FAs), which are bound by the cytoskeleton and heterodimers of α- and β-integrins [9]. Cellular Fas, which form cellular systems that sense and respond to external physical and chemical signals [9], are flat and elongated structures 1–5 µm in length, 0.3–0.5 µm in width and ~0.05 µm in thickness. They are composed of various macromolecules forming focal adhesion complexes (FACs). These include: α- and β-integrin transmembrane receptors; intracellular adaptor proteins (IAPs), such as scaffold talin, vinculin and paxillin; and IAP-recruited signaling molecules, such as FAK and ras homolog gene (Rho) family GTPases, including RhoA, ras-related C3 botulinum-toxin substrate-1 (Rac1) and cell division-control protein-42 (Cdc42) [10]. Among these, FAK a tyrosine kinase, plays a central role in functioning as a scaffold for other focal adhesion components and as a critical signaling initiator for various pathways via its binding to Src, Cas and paxillin [11]. For example, FAK has been found to control the regulation of the mammalian target of rapamycin complex-1 (mTORC1), extracellular signal-regulated kinase-1/2 (ERK1/2) and NF-κB signaling [12,13]. In addition, FAK regulates RhoA activation [14] and controls OBD and bone regeneration [15].

We previously applied a random positional 3D clinostat to model SMG for the investigation of SMG’s effect on tumor cell biology and demonstrated that the SMG inhibited tumor cell proliferation, migration and invasiveness and the promotion of tumor cell apoptosis via FAK-regulated mTORC1, AMPK and ERK1/2 pathways [16,17]. Our data thus suggest that FAK may play a central role in various SMG-induced cellular alterations via distinctive pathways [18], possibly including SMG-inhibited OBD. Therefore, we hypothesized that FAK is an up-stream molecular signal regulating the Wnt/β-catenin pathway in the SMG-induced inhibition of OBD.

To assess our hypothesis, we investigated SMG’s effects on the modulation of OBD in experiments using a mouse osteoblast cell line, MC3T3-E1, along with 3D clinostat modeling. We demonstrated that the SMG altered the cytoskeleton structure, reduced the formation of FAs and down-regulated FAK signaling in the MC3T3-E1 cells cultured under SMG (µg), compared to the cells cultured under ground (1 g) conditions. In addition, the SMG also down-regulated the expression of the Wnt-regulated transcription factor β-catenin and the osteoblastic maturation markers BMP2 and COL1, as well as inhibiting ALP activity and mineralization. In a mouse hindlimb unloading (HU) model, we found reduced trabecular bone volume measured by micro-computed tomography (micro-CT) imaging and confirmed by histological analyses in the HU-treated mouse tibia, compared to the control mouse tibia. Interestingly, the FAK activator, cytotoxic necrotizing factor-1 (CNF1) [16,17], converted the above alterations in the cytoskeleton structures, FAs, various gene expressions, the OBD of SMG-treated MC3T3-E1 cells and the bone density of HU-treated mice.

## 2. Results

### 2.1. Simulated Microgravity Alters Cytoskeleton Structures and Reduces Focal Adhesions (FAs)

**Figure 1:**
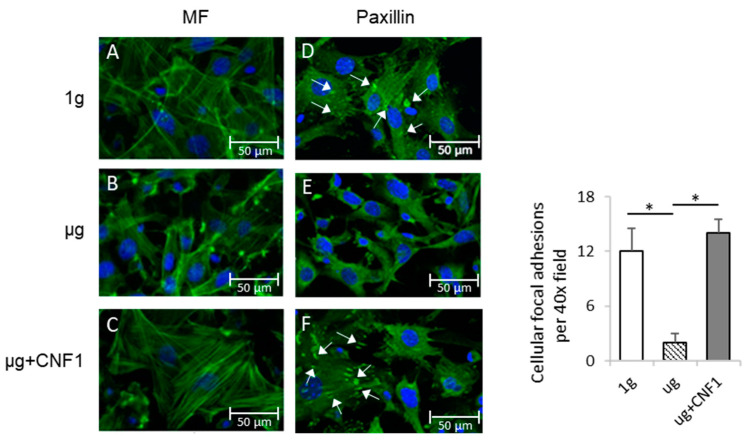
Measurement of cytoskeleton structures and focal adhesions of MC3T3-E1 cells cultured under different conditions. MC3T3-E1 cells were cultured in chamber slides under 1 g (**A**,**D**), μg (**B**,**E**) and μg + CNF1 (**C**,**F**). The cells on slides were stained with (**A**–**C**) FITC-phalloidin (green) and (**D**–**F**) anti-paxillin antibody (green). Slides were then covered with cover slips using Prolong Gold Antifade Reagent with DAPI (blue) and analyzed for microfilament (green) and focal adhesions (green), respectively, by fluorescence microscopy using 40× objectives (formation of cellular focal adhesions, white arrows). The average numbers of cellular focal adhesions per 40× field were measured by using ImageJ software. Data represent the mean ± SD. * *p* < 0.05 versus different groups by using Student *t* test. One representative experiment of two is shown.

To assess the SMG-affected cytoskeleton structures and cellular FAs, we stained MC3T3-E1 cells cultured in chamber slides coated with integrin ligand fibronectin under SMG (µg) and ground (1 g) conditions for 3 days with fluorescein isothiocyanate (FITC)-labeled phalloidin and FITC-labeled anti-integrin-binding protein paxillin antibody. This was followed by an examination of the cells by fluorescence microscopy for the measurement of the microfilament structure and FAs, respectively. We demonstrated that the osteoblastic cells cultured under 1 g condition showed abundant stress fibers (actin/myosin bundles) (Figure 1A), while the cells under µg conditions showed a dramatic decrease in stress fibers (Figure 1B). We also found that the FA (paxillin) spots (white arrows) were substantially reduced in the MC3T3-E1 cells cultured under SMG (Figure 1D), compared to the control MC3T3-E1 cells cultured under 1 g conditions (Figure 1E). These data indicate that SMG dramatically alters cytoskeleton structures and reduces the formation of cellular FAs, which is consistent with our previous reports [16,17].

### 2.2. Simulated Microgravity Inhibits FAK Signaling

**Figure 2:**
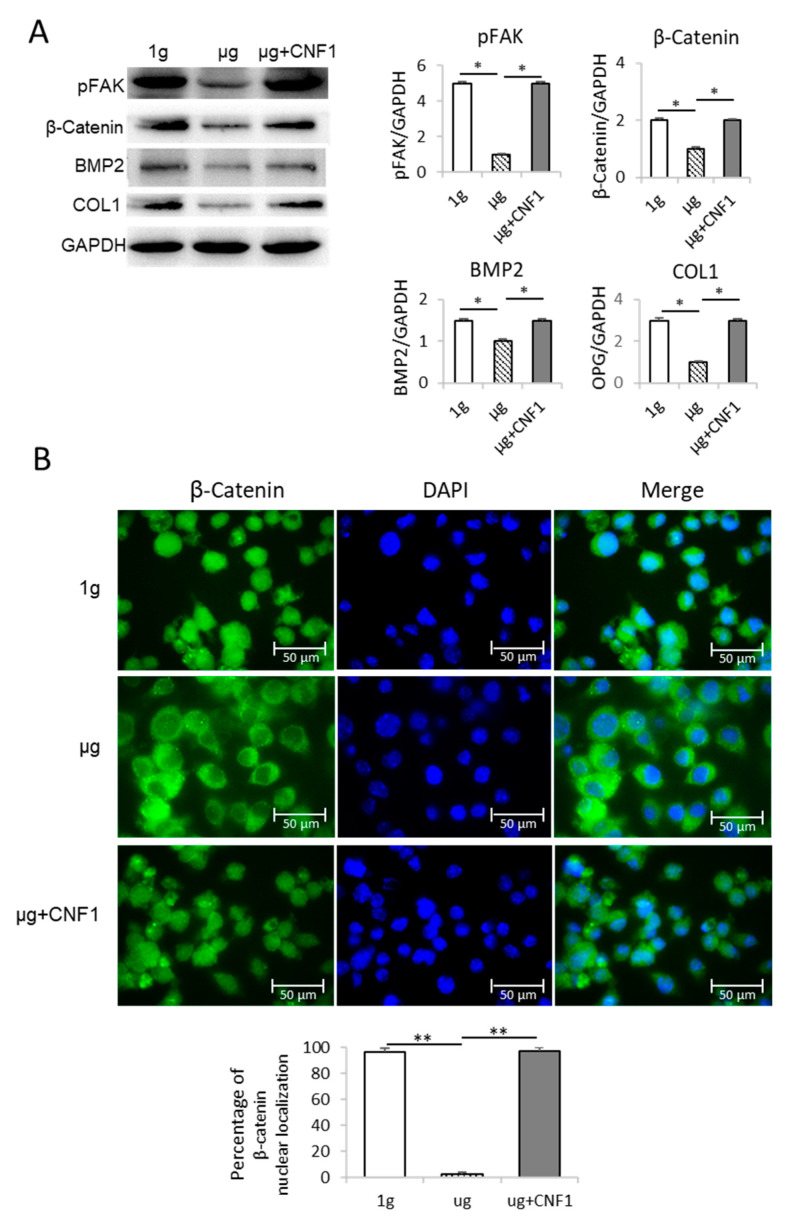
Analysis of FAK and Wnt/β-catenin-regulated gene signaling and β-catenin nuclear localization in MC3T3-E1 cells cultured under different conditions. (**A**) Cell lysates prepared from MC3T3-E1 cells cultured in flasks under 1 g, μg and μg + CNF1 were subjected to SDS-PAGE analysis, respectively. Proteins were transferred onto PVDF membranes and blotted with indicated antibodies. Western blot band signals were quantified by chemiluminescence. Densitometric values were normalized for matching GAPDH controls. Data represent the mean ± SD of three replicates. * *p* < 0.05 versus different groups by Student *t* test. (**B**) β-catenin nuclear localization analysis. MC3T3-E1 cells grown on chamber slides were covered using Prolong Gold Antifade Reagent with DAPI (blue) and observed by confocal microscopy. The percentages of β-catenin nuclear localization were measured by using ImageJ software. Data represent the mean ± SD. ** *p* < 0.01 versus different groups by using Student *t* test. One representative experiment of two is shown.

To investigate SMG-affected FAK signaling, we performed a Western blotting analysis using cell lysates derived from MC3T3-E1 cells cultured in a culture medium under µg and 1 g conditions, as described in the Materials and Methods section. We demonstrated that the SMG significantly reduced the abundance of phosphor FAK (pFAK; Y397) in the cells under µg compared to the cells cultured under 1 g conditions (Figure 2A), indicating that SMG down-regulates FAK signaling.

### 2.3. Simulated Microgravity Suppresses Expression of Wnt/β-catenin, BMP2 and COL1

To assess the SMG-affected Wnt’s down-stream molecules, we performed a Western blotting analysis using cell lysates derived from the MC3T3-E1 cells cultured under µg and 1 g conditions for 3 days. We found that the SMG also significantly reduced the expression of the Wnt-regulated transcription factor β-catenin and the osteoblastic markers COL1 and BMP2 [19] in the MC3T3-E1 cells cultured under µg, compared to the MC3T3-E1 cells cultured under 1 g conditions (Figure 2A), indicating that SMG inhibits OBD by suppressing the Wnt pathway. It has been reported that active β-catenin molecules transported into the nucleus stimulate the expression of its down-stream transcription factors TCF1 and LEF in the nucleus for OBD [7]. To measure the β-catenin localization, the MC3T3-E1 cells cultured under µg and 1-g conditions were stained with DAPI for nuclear (blue) and FITC-labeled anti-β-catenin antibody for β-catenin (green), and then measured by confocal microscopy [20,21]. We demonstrated that significantly more β-catenin molecules were found in the nuclei of the MC3T3-E1 cells cultured under 1 g condition compared to the MC3T3-E1 cells under µg condition (Figure 2B).

### 2.4. Simulated Microgravity Reduces ALP Activity and Matrix Mineralization

**Figure 3:**
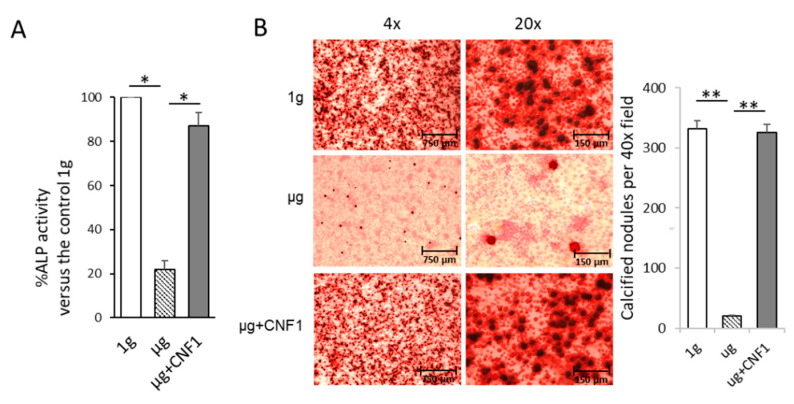
Assessment of ALP activity and mineralization in MC3T3-E1 cells cultured under different conditions. (**A**) Lysates of MC3T3-E1 cells cultured in flasks under 1 g, μg and μg + CNF1, respectively, were subjected to ALP activity analysis. Data represent the mean of a triplet ± SD in three experiments. * *p* < 0.05 versus different groups by Student *t* test. (**B**) MC3T3-E1 cells cultured in chamber slides under 1 g, μg and μg + CNF1, respectively. The cells on slides were fixed with 4% paraformaldehyde and then stained for 30 min with Alizarin red at 37 °C, followed by measurement of Alizarin-red-stained calcified nodules (mineralization) by light microscopy. The calcified nodules per 40× field were measured by using ImageJ software. Data represent the mean ± SD. ** *p* < 0.01 versus different groups by using Student *t* test. One representative experiment of two is shown.

Osteoblasts produce ALP, which is a critical component for the formation of hydroxyapatite and the calcification and mineralization of bones [22]. We therefore measured two osteoblast maturation characteristics, ALP activity and mineralization [19], by using the lysates of the MC3T3-E1 cells cultured under 1 g and µg conditions for ALP activity analysis and staining the MC3T3-E1 cells cultured under 1 g and µg conditions with Alizarin red for light microscopy analysis, respectively. We demonstrated that the SMG significantly reduced the ALP protein level and mineralization in the MC3T3-E1 cells cultured under µg, compared to the MC3T3-E1 cells cultured under 1-g conditions, respectively (Figure 3A,B). Altogether, our data demonstrate that SMG inhibits FAK and Wnt/β-catenin signaling, down-regulates Wnt/β-catenin’s down-stream molecules (BMP2 and COL1) for OBD and suppresses MC3T3-E1 cells’ ALP activity and matrix mineralization.

### 2.5. CNF1 Restores SMG-Induced Alterations in Cytoskeleton Structure, Focal Adhesions, Gene Expression and Osteoblast Differentiation/Maturation

The *E. coli* toxin, CNF1, is a broad-spectrum activator of FAK and RhoA signaling [16,17,23]. To assess whether the activation of FAK stimulates the SMG-inhibited Wnt/β-catenin pathway, we performed similar experiments by using MC3T3-E1 cells cultured under µg and µg + CNF1 (30 ng/mL), followed by various cellular analyses, as described above, and as we described in a previous paper [16,17]. Interestingly, we demonstrated that CNF1 (i) restored cytoskeleton structures and FA formation (Figure 1C,F), (ii) up-regulated FAK signaling, Wnt-controlled transcription factor β-catenin and the osteoblast maturation markers BMP2 and COL1 (Figure 2A), (iii) enhanced β-catenin localization in the nucleus (Figure 2B), and (iv) activated the osteoblast maturation characteristics, ALP activity and matrix mineralization, in the MC3T3-E1 cells cultured under µg conditions (Figure 3). Altogether, our data indicate that FAK is a critical upstream signal controlling Wnt/β-catenin-regulated OBD.

### 2.6. HU Induces Bone Density Reduction and Bone Loss in HU-Treated Mice

**Figure 4:**
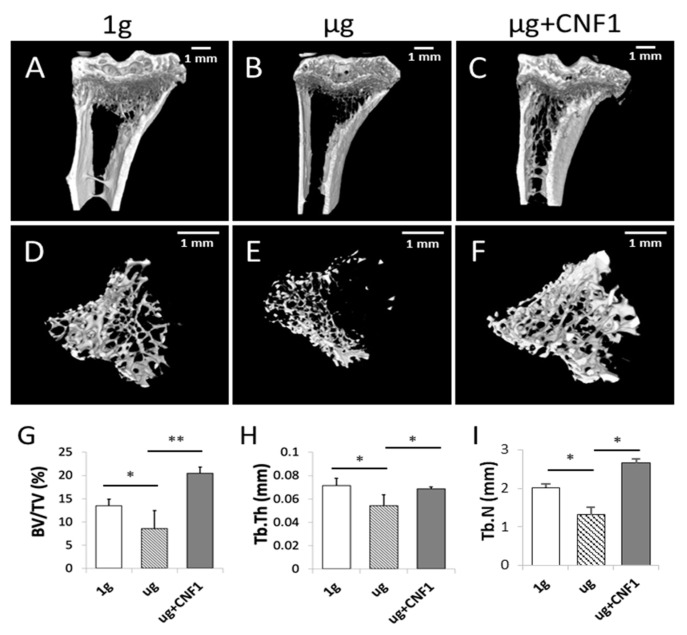
Measurement of mouse proximal trabecular structures by micro-CT imaging in HU-treated (*n* = 5), HU + CNF1-treated (*n* = 4) and control (*n* = 8) mice. (**A**–**F**) Micro-CT showed that HU-treated mice had significantly less trabecular bone formation than the control mice and that HU+CNF1-treated mice effectively converted HU-inhibited trabecular bone formation. (**G**–**I**) Bone volume fraction (BV/TV), trabecular thickness (Tb.Th) and trabecular number (Tb.N) all significantly decreased in tibias of HU-treated mice compared to control mice, and activation of FAK restored HU-affected trabecular bone formation. Scale bar: 1 mm. Data represent the mean ± SD. * *p* < 0.05 and ** *p* < 0.01 versus different groups by Student *t* test.

**Figure 5:**
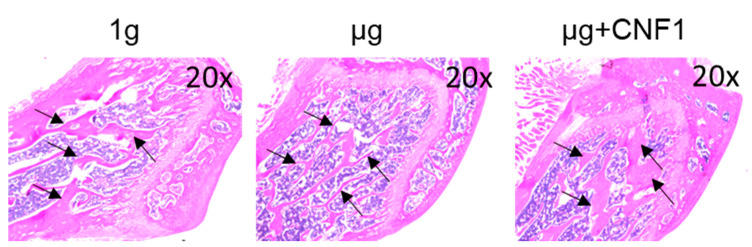
Measurement of mouse proximal trabecular structures by histology in HU-treated (µg), HU+CNF1-treated (µg + CNF1) and control (1 g) mice. Compared to the control group, H/E bone sections of HU-treated (µg) mouse tibias showed significantly thinner trabecular structures than control (1 g) mice; and administration of CNF1 converted HU-affected trabecular bone formation in HU-CNF1-treated (µg + CNF1) mice. Black arrows represent trabecular bone structures. Magnification: 20×. One representative bone sample of three to four is shown.

Mouse hindlimb unloading (HU) modeling is a well-tolerated approach mimicking the removal of weight-bearing loads (i.e., AMG or µg) during spaceflights that has been widely used to study SMG-induced bone loss in vivo [24,25]. It has recently been demonstrated that HU-induced bone loss is also controlled by the inhibition of the Wnt/β-catenin pathway [26]. To assess the HU-affected bone loss, we performed HU experiments on mice, followed by the collection of mouse-tibia bone samples for micro-CT imaging and histological analyses. The micro-CT images of the proximal tibial epiphysis and metaphysis were reconstructed in three dimensions to assess the structural details of the trabecular bones. The HU tibias presented with a typical osteopenia phenotype, which was characterized by sparse fractured trabecular architecture (Figure 4B,E), compared to the control tibias (Figure 4A,D). The analyses of trabecular structures within the proximal tibia with micro-CT displayed a significant reduction in bone volume fraction (BV/TV), trabecular thickness (Tb.Th) and trabecular number (Tb.N) in the bone samples derived from the HU-treated mice, compared to those of the control mice (Figure 4G–I). Our data indicate that the HU modeling induced a significant loss of trabecular bone in the HU-treated bone samples, as measured by the micro-CT imaging analysis, which was further confirmed by the data showing thinner trabecular bone structures in the H/E sections of the proximal tibia specimens in the HU-treated (µg) mice, compared to the control (1 g) mice without any treatment, according to the microscopy analysis (Figure 5).

### 2.7. CNF1 Rescues HU-Induced Bone Density Reduction and Bone Loss

The *E*. *coli* toxin CNF1 is a well-tolerated pharmaceutical reagent that has been used as a pioneering therapy for central nervous system diseases [23]. To assess whether the CNF1 prevented bone loss in the HU-treated mice, we collected tibia bone samples derived from the mice treated with HU and HU+CNF1 (3 weeks), respectively, followed by micro-CT imaging and histological analyses. We demonstrated that FAK activation through the oral administration of CNF1 (10 µg/each dose, for a total of four doses, during 3 weeks of HU) effectively restored the HU-induced trabecular bone loss, as measured by the micro-CT imaging (Figure 4C,F–I) and the histological analysis (Figure 5).

## 3. Discussion

**Figure 6:**
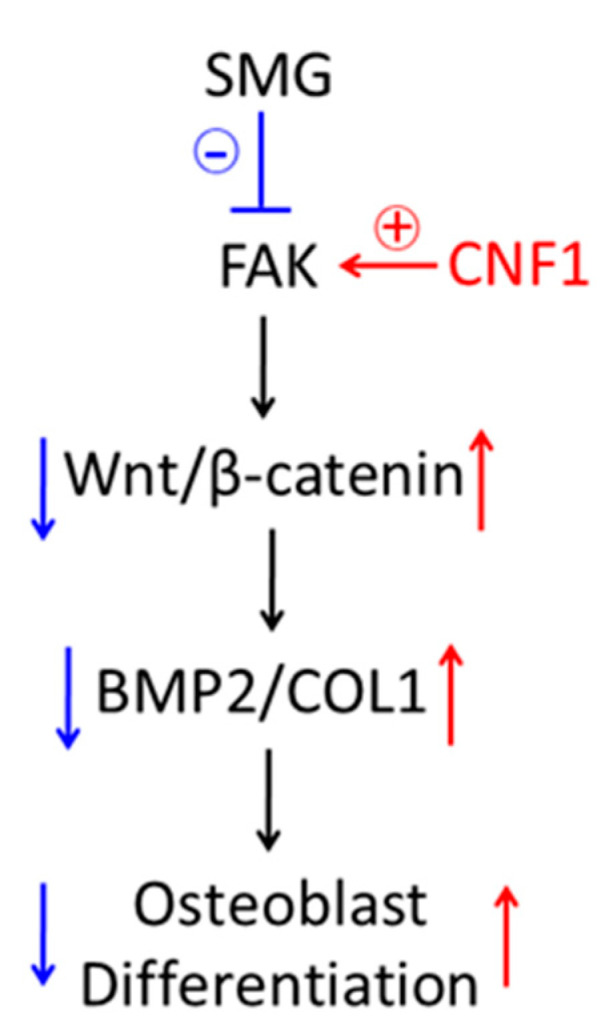
Schematic diagram presenting how SMG inhibits focal adhesions and down-regulates FAK signaling, leading to inhibition of osteoblast differentiation and bone loss via Wnt/β-catenin pathway, and activation of FAK signaling by CNF1-converted, SMG-affected osteoblast differentiation and bone loss via Wnt/β-catenin pathway.

In this study, we investigated SMG’s effects on the modulation of OBD in experiments using a mouse osteoblast cell line, MC3T3-E1, along with the 3D clinostat modeling SMG condition. We demonstrated the following: (i) SMG alters cytoskeleton structures, inhibits FA formation and down-regulates FAK signaling and the Wnt/β-catenin-BMP2/COL1 pathway controlling OBD, while mouse HU modeling mimicking the AMG condition also reduces trabecular bone volume in the proximal tibia through the thinning and loss of trabeculae; and (ii) the activation of FAK by the activator CNF1 successfully converts SMG-induced alterations in cytoskeleton structure, FAs, various gene expressions, ALP activity and matrix mineralization in MC3T3-E1 cells cultured under µg conditions and prevents bone loss in HU-treated mice. Therefore, our data indicate that (i) FAK is an upstream signal regulating Wnt/β-catenin-BMP2/COL1 pathway regulating OBD and (ii) FAK activation restores the SMG-induced inhibition of OBD via the Wnt/β-catenin pathway (Figure 6).

The *E. coli* toxin, CNF1, is a 114 kDa single-chain multidomain protein that contains an N-terminal receptor-binding domain, a translocation domain and a C-terminal catalytic domain [23]. After binding to the 67 kDa form of the laminin receptor precursor, CNF1 is internalized and transferred into cytosols, where it catalyzes a reversible activation of the Rho family GTPases [23] controlling gene transcription, actin cytoskeleton organization, cell proliferation, migration and survival [27]. CNF1 has been shown to play an important role in neuronal remodeling, improve memory performance, decrease β-amyloid accumulation and control neuroinflammation in murine models of Alzheimer’s disease [23,28].

In addition to the Wet/β-catenin pathway, some other molecular pathways, such as phosphoinositol 3-kinase (PI3K)-AKT-mTORC1, ERK1/2 and AMPK, have also been reported to regulate OBD [29,30,31]. FAK is a critical signaling initiator for various pathways [11], such as mTORC1 and ERK1/2 [12,13]. FAK also controls OBD and bone regeneration [15]. We previously demonstrated that SMG inhibited tumor cell proliferation, migration and invasiveness, as well as promoted tumor cell apoptosis via the FAK-regulated mTORC1, AMPK and ERK1/2 pathways [16,17]. Therefore, we assume that the activation of FAK may also convert the SMG-induced inhibition of OBD and that bone loss could be derived from combinational pathways, including not only the Wnt/β-catenin, but also the mTORC1, AMPK and ERK1/2 pathways. To test this assumption, an assessment of the conversional effect of CNF1 on MC3T3-E1 cells with each of the above pathways blocked, using siRNA-targeted lentiviruses to silence Wnt/β-catenin, mTORC1, AMPK and ERK1/2, respectively, is underway in our laboratory.

The fact that the Wnt/β-catenin pathway influences stem cell proliferation, differentiation and maintenance makes it an attractive target for the development of pharmaceutical agents to treat cancer, neurological disorders and bone repair/regeneration [32]. In addition to blocking the Wnt/β-catenin pathway with small molecules for cancer and neurological diseases [33], targeting the Wnt/β-catenin pathway could also lead to therapeutic agents aimed at stimulating the pathway for bone repair in clinic osteoporosis [32]. The Wnt protein’s hydrophobicity makes it difficult to produce in a sufficient quantity to make it a feasible therapeutic reagent. However, small molecules intervening in different compounds may overcome this problem and offer therapeutic potential [3]. One of these molecules is lithium, which has promising effects on bone healing and inhibits GSK3β, thereby activating Wnt/β-catenin [34,35]. Although the therapeutic effect of lithium in promoting bone healing has been well documented in experimental settings and clinical trials, its major medical limitation is still its clinical underutilization, which is attributable to its unfavorable side effects [36]. Therefore, searching for new drugs with efficient activation of the Wnt/β-catenin pathway, but with fewer side effects, is warranted.

Insulin-like growth factor-1 (IGF1), the most abundant growth factor in the bone matrix, plays an important role in the stimulation of OBD and bone regeneration through its binding to IGF1 receptor (IGF1R) [37]. The binding of IGF1 to IGF1R triggers various substrates, such as insulin receptor substrate-1 (IRS1), Src homology and collagen protein (SHC) [37], leading to IRS1’s activation of the PI3K-AKT-mTORC1 pathway and SHC’s activation of the ERK1/2 pathway [38], as well as OBD and bone regeneration [39]. IGF1 significantly promotes OBD [38,39] and increased the bone density of rodents both in HU modeling and in a space shuttle [40,41]. Clinical trials have been conducted on the effect of the administration of recombinant IGF1 on bone healing [42,43]. Notably, IGF1 has also been found to promote adipogenic differentiation [44]. In addition to IGF1-induced side effects [42], some contradictory studies showing no effect on bone density from IGF1 treatment have also been published [45]. Therefore, the question of how to enhance bone density and regeneration by IGF1 remains of great interest amongst bone biologists, as well as medical doctors.

Compared to lithium’s activation of the Wnt/β-catenin pathway and IGF1’s activation of both the mTORC1 and ERK1/2 pathways, we assume that the FAK activator CNF1’s activation of the Wnt/β-catenin, mTORC1, ERK1/2 and AMPK pathways may be the most efficient for the conversion of the SMG-induced inhibition of OBD and HU-induced bone loss. To assess our assumption, we are in the process of conducting experiments using in vitro MC3T3-E1 cells cultured in a medium that includes CNF1, lithium and IGF1, respectively, under 3D clinostat-induced SMG conditions, as well experiments using in vivo HU-treated mice with the administration of CNF1, lithium and IGF1, respectively.

The use of novel gene-targeted therapeutics has become a new direction of drug development [46]. The ground-based SMG system that has served to advance our understanding of the fundamental role of AMG in cellular and molecular biology can serve as a novel platform for new drug discovery [47]. The E. coli toxin, CNF1, is a well tolerated pharmaceutical reagent that has been used as a pioneering therapy for the central nervous system diseases in rodents [23,48]. We previously demonstrated that CNF1 also activates FAK; the activation of FAK converts the SMG-altered tumor cell proliferation and metastases via the mTORC1 and AMPK pathways [16] and restores SMG-promoted cell apoptosis via the mTORC1/NF-κB and ERK1/2 pathways [17]. Using the SMG platform, we have provided the first evidence that the activation of FAK by CNF1 converts SMG-induced OBD inhibition and prevents bone loss in HU-treated mice. Further research is warranted to confirm the CNF1-activated FAK-induced conversional effect on the SMG-induced inhibition of OBD using human osteoblast cells cultured under AMG condition as well as the replication of HU-induced mouse bone loss in mice hosted on spacecraft.

Altogether, we have demonstrated that FAK signaling plays a critical role in SMG-induced OBD inhibition via the Wnt/β-catenin pathway and that the activation of FAK by CNF1 successfully converts SMG-induced OBD inhibition and prevents bone loss in HU-treated mice. Therefore, the use of CNF1 modulating multiple FAK-regulated pathways for bone regeneration, including the Wnt/β-catenin pathway, may become a new target in the development of novel therapeutics for astronauts at risk of OBD inhibition and bone loss, and for clinical patients with osteoporosis.

## 4. Materials and Methods

### 4.1. Cells, Antibodies and Reagents

A mouse osteoblast cell line, MC3T3-E1, obtained from Thermo Fisher Scientific (Rockford, IL, USA) was maintained in a regular α-MEM medium plus 10% fetal calf serum (FCS). Fluorescein isothiocyanate (FITC)-labeled and rabbit antibodies against paxillin were purchased from Thermo Fisher Scientific. Primary rabbit antibodies against phosphor-FAK (pFAK; Y397), β-catenin, BMP2, COL1 and glyceraldehyde 3-phosphate dehydrogenase (GAPDH) were obtained from Cell Signaling Technology (Boston, MA, USA). FITC-conjugated goat anti-rabbit (ZF-0314) secondary antibody specific for primary antibodies were purchased from Thermo Fisher Scientific. The Prolong Gold Antifade Reagent with DAPI was obtained from Life Technologies Inc. (Carlsbad, OTT, Canada). Ascorbic acid, β-glycerol phosphatase and Alizarin red were obtained from Sigma-Aldrich (St. Louis, MO). *E. coli* toxin cytotoxic necrotizing factor-1 (CNF1), which activates FAK and RhoA [16,17,23], was obtained from Dr. Harald Genth, Hannover Medical School, Hannover, Germany [49].

### 4.2. Clinostat of Simulated Microgravity and Cell Culture

SMG is modeled by the random positional machine (RPM), which is a three-dimensional (3D) clinostat manufactured by Center for Space Science and Applied Research, Chinese Academy of Sciences (Beijing, China) [16,17]. The RPM consists of two independent rotating frames, an inner frame and an outer frame. Both frames can randomly rotate in 3D with changes in acceleration and direction of the samples over time, resulting in randomization of the gravitational vector, low fluid shear stress and 3D spatial freedom. The angular velocity of the rotation is at a speed of 30°/s. To assess SMG’s effect on osteoblast differentiation, MC3T3-E1 cells were grown in a Lab-Tek II Chamber SlideTM System (Nalge Nunc International Inc., Rochester, NY, USA) or T25 culture flasks, which were filled with osteoblast maturation medium [regular medium supplemented with ascorbic acid (2.5 µg/mL), β-glycerol phosphate (10 mM) and dexamethasone (0.1 µM)] [50] to avoid the presence of air bubbles for prevention of fluid shear stress, sealed and placed on a clinostat under µg conditions at 37 °C in a CO_2_ incubator. Since it takes more than two weeks for MC3T3-E1 cell maturation to form mineralization [51,52] and MC3T3-E1 cells often become detached from culture flasks during cell culturing under µg condition, we adopted an incubation procedure of 6 h/day at 37 °C under µg conditions for 12 days for matrix mineralization analyses and, subsequently, for 3 days for Western blot, fluorescent/confocal microscopy or ALP activity analyses [19]. This cell incubation procedure with a shorter daily exposure to SMG has been shown to provide similar microgram-induced effects on osteoblast gene expression, ALP activity and mineralization compared to the cell incubation protocol with a full-time daily exposure to AMG on space stations [53]. The control cells were placed in the incubator under 1-g conditions. CNF1 has been extensively used to treat mammalian cells in cell cultures at concentrations ranging between 10 and 100 ng/mL for measurement of CNF1’s effect on molecular pathway modulations [54,55,56]. To assess conversional effect of CNF1 on SMG, we chose to add an intermediate dose of CNF1 (30 ng/mL) to the above MC3T3-E1 cells cultured under SMG, as we previously described [16,17].

### 4.3. Fluorescent Microscopy

For microtubule immunofluorescence staining, MC3T3-E1 cells cultured in chamber culture slides were fixed in 4% paraformaldehyde for 15 min at room temperature and permeabilized using PBS containing 0.5% Triton X-100, followed by blocking in 1% bovine serum albumin (BSA) in PBS for 30 min at room temperature. To check microfilaments, the cells were incubated with 1:20 FITC-labeled phalloidin diluted in PBS for 30 min in dark at room temperature. To measure cell focal adhesions, the cells were incubated with rabbit anti-paxillin antibody (1:100 diluted in PBS) containing 1% BSA for 24 h at 4 °C overnight, followed by 1:100 FITC-labeled anti-rabbit antibody (green). Plastic chambers were removed after rinsing three times with PBS. The slides were covered with cover slips and then measured for microfilament structure and focal adhesions, respectively, by fluorescence microscopy [16,17].

### 4.4. Western Blotting Analysis

MC3T3-E1 cells cultured in flasks were lysed in lysis buffer containing 1% NP40, 0.5% sodium deoxycholate, 0.1% SDS in PBS containing protease and phosphatase inhibitors, for 30 min, on ice. The cell lysates were centrifuged at 4 °C for 30 min at 12,000 rpm and protein contents in the supernatant were quantified by using BCA Protein Assay Kit (Thermo Fisher Scientific, Waltham, MA). For Western blotting analysis, 30–50 μg total protein sample in the supernatant was loaded into each well of 5–10% SDS-PAGE gradient gel. After electrophoresis, protein samples were transferred onto a 0.22-micrometer polyninylidene fluoride (PVDF) membrane (Millipore, Middlesex County, MA). Membranes blocked with 5% skimmed milk powder in TBST (pH 7.4, TBS with 0.1% Tween-20) were incubated with primary antibodies against various molecules overnight at 4 °C followed by incubation with suitable horseradish peroxidase-labeled secondary antibodies. The protein bands developed with horse radish peroxidase developer solution were quantified using chemiluminescence [16,17]. The GAPDH protein was used as an internal reference marker.

### 4.5. Confocal Microscopy

MC3T3-E1 cells cultured in Lab-Tek1 II chamber slides were permeabilized in PBS containing 0.5% Triton X-100 for 10 min, followed by blocking in 1% BSA for 30 min at room temperature. The cells were then incubated with FITC-rabbit anti-β-catenin antibody (green) (1:100) for staining of β-catenin in 1% BSA for 2 h in dark at room temperature. After rinsing cells three times with PBS, the slides were covered with Prolong Gold Antifade Reagent with DAPI (blue) for staining of nuclei and checked by confocal microscopy [20,21].

### 4.6. ALP Activity Assay

To measure ALP activity, we performed in vitro experiments using the protein samples of MC3T3-E1 cell lysates and ALP activity assay kit (Abcam, Cambridge, MA, USA) according to the manufacturer’s manual [19]. The absorbance of samples in each well of a 96-well plate was determined at an OD of 405 nm on a Bio-Rad Model 3550 microplate reader. Relative ALP activity in experimental µg or µg + CNF1 sample was normalized to the cellular protein content measured using the bicinchoninic acid (BCA) Protein Assay Kit (Abcam, Cambridge, MA, USA) and presented as a percentage of ALP activity in control 1-g sample [50].

### 4.7. Mineralization

MC3T3-E1 cells cultured in Lab-Tek1 II Chamber Slides were fixed with 4% paraformaldehyde and then stained for 30 min with 0.5% Alizarin red (pH 4.2) at 37 °C. Alizarin red-stained calcified nodules on slides were measured by light microscopy [19].

### 4.8. Hindlimb Unloading

Mouse hindlimb unloading (HU) modeling has been widely used to mimic SMG condition for studying microgravity-affected alterations in mouse organs or tissues, such as brains and bones [24,25]. Briefly, each male BALB/c mouse (6–8 weeks) was individually housed in a cage and suspended by the end of its tail covered with a strip of adhesive surgical tape and attached to a chain hanging from a pulley. The mouse was suspended for 3 weeks at ~30° angle to the floor with only its forelimbs touching the floor such that the mouse could freely move and access food and water. Control mice were individually housed in cages without HU. To assess conversional effect of CNF1 on HU, HU-treated mice were orally administered CNF1 (10 µg/Kg mouse body weight) [57] in 200 µL H_2_O on the day for HU was started and, subsequently, once every six days for a total of four times, during three weeks of HU treatment period.

### 4.9. Micro-CT Imaging

The proximal tibia of each mouse was scanned using a SkyScan 1172 desktop micro-computed tomography (micro-CT) scanner (SkyScan, Kontich, Belgium) operating at 40 kVp and 250 µA, with 150-millisecond exposure times, 0.2 rotation step, four-frame averaging and a 0.5-mm aluminum filter at a voxel size of 10 µm. SkyScan’s NRecon software was used to reconstruct 3D datasets for analysis of trabecular bone microarchitectural parameters. All versions and subsequent analyses were performed using CT-Analyzer software. Bone volume fraction (BV/TV), trabecular thickness (Tb.Th) and trabecular number (Tb.N) were calculated by 3D standard microstructural analysis [58].

### 4.10. Histology

The bone samples were fixed using 4% paraformaldehyde, followed by decalcification using 10% ethylenediaminetetraacetic acid and embedding in paraffin for preparation of tissue sections. Hematoxylin/eosin (H/E)-stained tissue sections were checked by light microscopy.

### 4.11. Statistical Analysis

Statistical analysis was conducted using Graphpad Prism-3.0, and statistical significance among groups was analyzed using Student *t* test. Any *p* values <0.05 or <0.01 were considered statistically significant or very significant.

## Data Availability

Data are contained within the article.

## Abbreviations

ALP	alkaline phosphatase
AMG	aerospace microgravity
AMPK, AMP	activated protein kinase
BMP2	bone morphogenic protein-2
BSA	bovine serum albumin
CNF1	cytotoxic necrotizing factor-1
COL1	type-1 collagen
ERK1/2	extracellular signal-regulated kinase-1/2
FAK	focal adhesion kinase
FA	focal adhesion
FACs	focal adhesion complex
FITC	fluorescein isothiocyanate
GAPDH	glyceraldehyde 3-phosphate dehydrogenase
GSK3β	glycogen synthase kinase-3β
HU	hindlimb unloading
IAP	intracellular adaptor protein
IGF1	insulin-like growth factor-1
IGF1R, IGF1	receptor
LEF	lymphoid enhancer binding factor
Micro-CT	micro-computed tomography
mTORC1	mammalian target of rapamycin complex-1
OBD	osteoblast differentiation
pFAK	phosphor FAK
PVDF	polyninylidene fluoride
SMG	simulated microgravity
TCF1	T cell factor-1
